# Older Adults’ Experiences of a Physical Activity and Sedentary Behaviour Intervention: A Nested Qualitative Study in the SITLESS Multi-Country Randomised Clinical Trial

**DOI:** 10.3390/ijerph18094730

**Published:** 2021-04-29

**Authors:** Nicole E. Blackburn, Mathias Skjodt, Mark A. Tully, Ilona Mc Mullan, Maria Giné-Garriga, Paolo Caserotti, Sergi Blancafort, Marta Santiago, Sara Rodriguez-Garrido, Gudrun Weinmayr, Ulrike John-Köhler, Katharina Wirth, Javier Jerez-Roig, Dhayana Dallmeier, Jason J. Wilson, Manuela Deidda, Emma McIntosh, Laura Coll-Planas, on behalf of the SITLESS Group

**Affiliations:** 1 Institute of Nursing and Health Research, School of Health Sciences, Ulster University, Newtownabbey BT37 0QB, UK; ne.blackburn@ulster.ac.uk; 2 Department of Sports Science and Clinical Biomechanics, Center for Active and Healthy Ageing (CAHA), University of Southern Denmark, 5230 Odense M, Denmark; mskjodt@health.sdu.dk (M.S.); pcaserotti@health.sdu.dk (P.C.); 3 Institute of Mental Health Sciences, School of Health Sciences, Ulster University, Newtownabbey BT37 0QB, UK; m.tully@ulster.ac.uk (M.A.T.); i.mcmullan@ulster.ac.uk (I.M.M.); jj.wilson@ulster.ac.uk (J.J.W.); 4 Department of Sport Sciences, Faculty of Psychology, Education and Sport Sciences Blanquerna, Universitat Ramon Llull, 08034 Barcelona, Spain; mariagg@blanquerna.url.edu (M.G.-G.); martasc12@blanquerna.url.edu (M.S.); 5 Department of Physical Therapy, Faculty of Health Sciences Blanquerna, Universitat Ramon Llull, 08025 Barcelona, Spain; 6 Fundació Salut i Envelliment (Foundation on Health and Ageing)-UAB, Universitat Autònoma de Barcelona, 08041 Barcelona, Spain; sergi.blancafort@uab.cat (S.B.); sara.rod.garrido@gmail.com (S.R.-G.); 7 Institute of Epidemiology and Medical Biometry, Ulm University, 89075 Ulm, Germany; gudrun.weinmayr@uni-ulm.de (G.W.); u.j.koehler@gmx.de (U.J.-K.); 8 Agaplesion Bethesda Clinic, Geriatric Research Unit Ulm University and Geriatric Center, 89073 Ulm, Germany; katharina.j.wirth@googlemail.com (K.W.); ddallmei@bu.edu (D.D.); 9 Research Group on Methodology, Methods, Models and Outcome of Health and Social Sciences (M3O), Faculty of Health Sciences and Welfare, University of Vic-Central University of Catalonia (UVIC-UCC), 08500 Vic, Spain; javier.jerez@uvic.cat; 10 Department of Epidemiology, Boston University School of Public Health, Boston, MA 02118, USA; 11 Sport and Exercise Sciences Research Institute, School of Sport, Ulster University, Newtownabbey BT37 0QB, UK; 12 Health Economics and Health Technology Assessment (HEHTA), Institute of Health and Well-Being (IHW), University of Glasgow, Glasgow G12 8RZ, UK; manuela.deidda@glasgow.ac.uk (M.D.); emma.mcintosh@glasgow.ac.uk (E.M.)

**Keywords:** exercise referral schemes, qualitative study, behaviour change, sedentary behaviour, physical activity, ageing

## Abstract

Background: The SITLESS programme comprises exercise referral schemes and self-management strategies and has been evaluated in a trial in Denmark, Spain, Germany and Northern Ireland. The aim of this qualitative study was to understand the implementation and contextual aspects of the intervention in relation to the mechanisms of impact and to explore the perceived effects. Methods: Qualitative methodologies were nested in the SITLESS trial including 71 individual interviews and 12 focus groups targeting intervention and control group participants from postintervention to 18-month follow-up in all intervention sites based on a semi-structured topic guide. Results: Overarching themes were identified under the framework categories of context, implementation, mechanisms of impact and perceived effects. The findings highlight the perceived barriers and facilitators to older adults’ engagement in exercise referral schemes. Social interaction and enjoyment through the group-based programmes are key components to promote adherence and encourage the maintenance of targeted behaviours through peer support and connectedness. Exit strategies and signposting to relevant classes and facilities enabled the maintenance of positive lifestyle behaviours. Conclusions: When designing and implementing interventions, key components enhancing social interaction, enjoyment and continuity should be in place in order to successfully promote sustained behaviour change.

## 1. Introduction

Randomised controlled trials (RCTs) are widely used for establishing evidence of the effectiveness of interventions, yet public health interventions are complex, posing specific challenges for RCTs to overcome [1,2]. The complexity resides in the variety of behaviours required by participants in the intervention, the groups or organisational levels targeted, the potentially large number of outcomes and the degree of flexibility permitted by the intervention [3], all of which are associated with emergent phenomena that are difficult to predict. All these factors mean that complex health interventions are often challenging to define and therefore reproduce [4]. This poses specific challenges to evaluation [2], such as the organisational and logistical difficulty of applying RCT methods to service or policy change, and the length and complexity of the causal chains linking the intervention with an outcome.

There is growing recognition that current methodologies to understand complex public health interventions often fall short of comprehensively explaining which components of the intervention are effective or to the degree which they are effective [1,2,5]. Complex interventions need to be understood in ways that are responsive to the intricacies of programmes, people and places [5] to fully inform the design of future effective interventions. Qualitative research and mixed-method approaches attempt to overcome the limits of measurement-based research by adding meaning and providing important insights into the evaluation of health interventions [2].

Qualitative research is particularly valuable for evaluating complex health interventions where the conduct of the intervention is mediated by human behaviour [6]. Qualitative research can provide insight into the contextual circumstances and perceived effects through the exploration of lived experiences of people, thus improving the transferability and delivery of interventions by providing a more detailed understanding of procedures and processes that influence the results of an evaluation [1,2]. Moreover, complex interventions are increasingly recognised as belonging to “open” systems in ways that make planned interventions and their surrounding context difficult to disentangle using conventional RCT designs [7]. Accordingly, process evaluation, including qualitative and quantitative methodology, has the potential to provide a deeper understanding of the processes involved in implementing an intervention, which can facilitate research translation and interpretation of the results.

The SITLESS project fulfils the definition of a complex intervention, comprising of different active components (i.e., structured exercise programme, one-to-one visit, group-based sessions, telephone follow-up) that allowed for tailoring to improve a range of short-term and long-term physical, social and psychological outcomes [8]. With this in mind, the need for comprehensive and effective evaluation of interventions targeting older adults is needed in order to understand the components that can influence positive behaviours and result in sustained behaviour changes. Accordingly, the SITLESS study comprises a clinical trial focused on assessing effectiveness (i.e., impact evaluation) with quantitative methods and, complementarily, a process evaluation conducted with mixed methods. Process evaluation combines qualitative and quantitative results to help support the interpretation of impact evaluation outcomes [8].

Therefore, the main aim of this qualitative study, as part of the process evaluation of the SITLESS clinical trial, was to understand the implementation of the SITLESS programme and contextual aspects of the intervention in relation to the mechanisms of impact and to explore the perceived effects.

## 2. Materials and Methods

### 2.1. Study Design

This qualitative study is part of the process evaluation of the SITLESS trial conducted according to the published process evaluation framework, the full details of which can be found in the published protocol [8]. The SITLESS study was a multi-country randomised clinical trial which investigated the ability of an enhanced exercise referral scheme (ERS), including self-management strategies (SMS), to reduce sedentary behaviour, increase physical activity and improve physical function (ERS + SMS group) compared to a traditional ERS (ERS group) and a control group (CTRL group) receiving healthy lifestyle advice in 1360 community-dwelling older adults. Table 1 summarizes the general information of the ERS and SMS interventions and the control group. The process evaluation is structured in line with the Medical Research Council framework for evaluating complex interventions [9], with the aim of assessing the fidelity and reach of the implementation, understanding the contextual aspects of each intervention site, evaluating the mechanisms of impact and exploring the perceived effects. In order to provide deeper insights and a greater understanding of the impact and effect of the SITLESS intervention, a range of qualitative methodologies was nested in the trial, including individual interviews and focus groups. Four countries (Denmark, Spain, Germany and Northern Ireland) conducted the qualitative research based on a standardised protocol and semi-structured questioning approach. The SITLESS study targeted community-dwelling men and women aged ≥65 years from across Denmark, Spain, Germany and Northern Ireland. The study design was approved by the Ethics and Research Committee of each intervention site: The Ethics and Research Committee of Ramon Llull University (Fundació Blanquerna, Spain), The Regional Committees on Health Research Ethics for Southern Denmark (University of Southern Denmark, Denmark), Office for Research Ethics Committees in Northern Ireland (ORECNI) (Queen’s University of Belfast) and the Ethical Review Board of Ulm University (Ulm, Germany) [10].

### 2.2. Sampling and Recruitment

A combination of purposeful sampling strategies was employed, recruiting participants of the 3 arms (SMS + ERS group, ERS group and control group) and timepoints in relation to the end of the intervention (postintervention, 12- and 18-month follow-up) according to gender and ethnicity, from a range of socioeconomic backgrounds and functional levels (especially considering those who were classified as frail and robust according to their physical performance battery score). A maximum variation sampling method was used as a strategy to select a small number of cases that maximised the diversity relevant to the research question [11]. Each qualitative procedure targeted a specific purposeful sample of participants from each of the 4 intervention sites (Odense, Barcelona, Ulm and Belfast) according to the characteristics previously specified [8].

### 2.3. Participants

Participation was voluntary and all participants provided informed consent before the start of the study. Further details regarding recruitment, study procedures and intervention components can be found in the study protocol [10]. Only participants who attended the majority of their intervention arm and were able to comment on their experience of the programme were invited to participate in the qualitative study. No participants refused to take part in this component. Focus groups and interviews were held between November 2016 and January 2019 and lasted between 20 and 75 min. Focus groups were conducted face-to-face with participants across all intervention sites at postintervention (SMS + ERS group, n = 58; ERS group, n = 24), and semi-structured interviews were carried out face to face with participants at postintervention (SMS + ERS group, n = 15; CTRL group, n = 6) and during the 12 -month (SMS + ERS group, n = 8; ERS group, n = 8; CTRL group, n = 7) and 18-month follow-up periods (SMS + ERS group, n = 8; ERS group, n = 8; CTRL group, n = 7) to explore the participants experiences of the intervention and perceived impact of the programme. The data presented include the following representation from each site: Denmark (n = 38, 45% of women, mean age: 76.9 y, age range: 66–86 y, 1 from an ethnic minority), Germany (n = 27, 48% of women, mean age: 72.6 y, age range: 66–87 y, 4 from an ethnic minority), Spain (n = 46, 70% of women, mean age: 73.3 y, age range: 65–91 y, 1 from an ethnic minority) and Northern Ireland (n = 39, 59% of women, mean age: 74.3 y, age range: 66–99 y, 0 from an ethnic minority). Details regarding the data collected at each timepoint across the 3 groups are presented in Table 2.

### 2.4. Interview Schedule

The interview schedule was developed by the research team and included specific questions addressing the research aims. The questions on context explored the role of the physical and social environment and personal circumstances and tried to explore how context affects implementation and perceived effects. Moreover, the semi-structured interviews and focus groups included questions about the participants perceptions of the implementation of the specific intervention elements at postintervention. Research questions mechanisms of impact (i.e., how the delivered intervention produces change) and perceived effects (i.e., which effects could be attributed to the intervention as perceived by the participants) were explored during the postintervention and follow-up data collection phases. The facilitators at each site were encouraged to explore the topics and probe responses while also allowing pauses to encourage reflection and additional insight [12]. An iterative approach was taken whereby the topic guide was reviewed and updated after each focus group and/or interview as necessary in order to better answer the research question and ensure an opportunity to understand the experiences of the participants in greater detail. All topic guides used are available as Supplementary Materials.

### 2.5. Analysis

All focus groups and interviews were audio-recorded, and data were transcribed verbatim in the original language and validated for accuracy of transcription against the audio files. Two independent researchers completed coding of the transcripts at each site in the original language (NB, IM, LCP, MGG, MS, PC, MS, JJ, SRG, GW, UJK). The framework method was applied to analyse these data [13]. The analysis was conducted in the following stages: Transcription, familiarisation with the interview, coding of the transcripts, developing a working analytical framework, applying the analytical framework, charting data into the framework matrix and interpreting the data [13]. We explored the differences and commonalities regarding gender, functional level, ethnicity and socioeconomic background next to the type of intervention arm and timepoint in relation to the end of the intervention. The initial coding was conducted by 2 independent researchers and the codes were translated into English. As a next step, a working analytical framework was developed by discussing the codes assigned and similarities and differences to achieve an agreement on a set of codes to establish an initial analytical framework. This framework was then refined by coding further manuscripts until no new codes were generated by the 2 independent researchers. Once all the data were coded the qualitative team into identified themes, subthemes and categories were noted where appropriate to summarise the main findings within the prespecified framework matrix.

## 3. Results

The findings for the intervention groups (SMS+ERS and ERS) are reported under the framework categories of context, implementation, mechanisms of impact and perceived effects. Findings that are specific to the SMS + ERS group are identified within the text. The matrix of findings for the SITLESS intervention groups are presented in Table 3, and the control group findings are presented in Table 4.

### 3.1. Intervention Group Findings (SMS + ERS and ERS Participants)

#### 3.1.1. Context

The overarching theme to emerge regarding context was related to the environmental and personal factors that influenced older adults’ experience of the intervention (SMS + ERS and ERS). Three subthemes were identified, including: (i) social environmental factors, (ii) physical environmental factors and (iii) personal factors. Within the subtheme of social environmental factors, participants discussed issues relating to support at home, caring responsibilities and peer support. In terms of support at home, the topics surrounding these issues related to the positive influence support at home that could encourage healthy behaviours. Participants spoke about having family members that motivated their new lifestyle choices, with some family members getting involved and joining them, for example, on walks, and taking more of an interest in their own lifestyle choices and the associated benefits of having a supportive environment at home.

*P17 “Well my daughter said to me ‘you’re going to keep it up, aren’t you?’ and of course I am but she was really glad that we were doing it.” F*

In addition, the positive influence of peer support was something that the participants stated positively influenced their engagement in the programme. They highlighted that the social networks they developed throughout the programme supported their participation and involvement, acknowledging the importance of the social support and connectedness on adherence to the programme, maintaining healthier behaviours and developing support networks and relationships.

*P6 “It’s been nice to interact with others and be part of a group.” M*

*P604 “Being in the group is much more pleasant than being alone at home.” F*

Conversely, some participants alluded to negative contextual factors that hindered their ability to be active or prevented them from being as active as they would have liked. Some participants described how their caring responsibilities such as for grandchildren, partners or spouses, had a significant influence on the time they could dedicate to exercise or physical activity. This was more prevalent across female participants. The common issues relating to this category were surrounding time constraints and managing competing priorities.

*P13 “One of the things that I actually found was it really helped me up there [referring to her head] mentally I found that this has really gave me something to get up and out of the house for and I think I’m more productive at home because of it… When I was caring for my husband with dementia, I had no time to think about what I wanted to do and I’m no longer caring for him but this really gave me something to focus on and allowed me to focus on me.” F*

Within the subtheme of personal environmental factors, participants discussed barriers to PA. Seasonal influences such as poor weather were highlighted:

*P10 “There is a big difference between my level of physical activity during the winter versus summer period and I think it is important to focus on the dark periods when it’s raining, you have to make sure that older adults have something to do.” M*

*P11 “During the bad weather I usually wouldn’t get out but having this centre has meant that I can keep it up without having to be outside walking.” F*

In terms of safety in their local neighbourhood, participants described barriers to being active due to fears for their personal safety and issues relating to their own physical and functional capabilities being on their own away from their homes. An interesting area of discussion with the participants was related to preconceptions of fitness centres and gyms. Participants described biases relating to the perception of people who attended fitness centres and gyms, which was not the case when they attended the settings. Participants described their surprise to see others of their own age using the facilities and their relief by how warm the staff and other members were toward them, making them feel welcomed in a setting that was outside of their comfort zone.

*P12 “I was so apprehensive and afraid of going on any of the machines, but it has been wonderful for me health-wise. I really enjoyed it, every minute of it.” F*

The subthemes of personal factors included issues relating to health and well-being, personality types and mood and the recognition of meaningful activity (i.e., exercises which improved their ability to conduct activities of daily living). Participants described the implications associated with their health and functional status that impacted on their engagement in the programme.

*P28 “My husband and I would have come down [a local walk] with the dogs and we loved that but now hills and things really restrict me. More recently as I’m now on my own, I’m even more restricted due my health and no longer driving has an impact on how much I get out and about.” F*

Issues relating to the impact of the different personality types were highlighted as both a barrier and facilitator to engagement in the programme within groups, with some participants commenting on the atmosphere of the session being dependent on those taking part in the sessions. Mood was also stated as something that could influence their experience of the programme. Participants noted effects of some medications and family issues or caring responsibilities that could affect their mood. The recognition of meaningful activity was stated as a facilitator to engagement in the programme. Participants described an appreciation in doing something that benefited them and made them feel better in themselves.

*P18 “Looking at my physical activity or lack of activity this was a real good opportunity to kick start me into doing a bit more activity.” M*

#### 3.1.2. Implementation

The overarching theme to emerge regarding implementation of the intervention was related to the participants’ views on the components of the SMS + ERS and ERS programme. Three subthemes emerged: (i) Social enablers, (ii) practical enablers and (iii) structural enablers. Within the subtheme of social enablers, participants discussed matters relating to the trainer, their peers and their own personal enjoyment and satisfaction with the programme. It was evident from the discussions that the personality of the trainer had a very prominent role in their experience of the programme, and this was something that participants reflected on strongly when asked to describe their experiences of the intervention.

*P32 “It is important to exercise at our age, but it is also important to have a trainer who can show you empathy. That is maybe the most important thing… our trainer had empathy and that was important for the social dynamics and why we continued with the exercise program.” F*

The group-based element of the exercise component and SMS sessions were highlighted as being a very encouraging aspect of the programme. Participants stated that the group-based physical activity created and strengthened feelings of connectedness, community and belonging.

*P21 “I really enjoyed that part and thought it was really good, doing it as a group. I just love people you know what I mean. Oh that reminds me, I must give [participant] a ring and see how she is. But yes, I loved being part of a group and getting to know people.” F*

In terms of peers, participants noted the benefits associated with exercising with people their own age and with similar abilities. They said they felt comfortable during the class and really enjoyed being part of the group. The participants commented on the positive influence of support and social connectedness on adherence.

*P14 “Yes, and you don’t feel out of place because you’re in with people of your own age group and you feel well… I know that I’m older than most of you [laughter] and before I came I thought I was active, I played golf twice a week and I played bowls twice a week but because I took pleurisy and pneumonia I couldn’t do those things so coming here gave me the incentive, I’m inside, I’m doing exercise and it really got me back into the swing of things again and for walking I would do quite a lot of walking and I would meet [participants] out walking but this has been really fantastic…” F*

Within the subtheme of practical enablers, participants from both intervention groups spoke positively of the facilities in which the programme was held. These facilities varied across the four intervention sites: Some countries conducted sessions outdoors, while others were mainly held in indoor gym facilities. Some of the indoor sites were of a high specification and included a range of facilities that the participants could utilise while involved in the programme, for example, swimming pools and changing and shower facilities. In some sites, participants stayed after the classes for a cup of tea or coffee and some social time with other participants at the end of their session. The staff within the facilities was also highlighted as having a positive impact on their experience. The participants commented on how they were welcomed and greeted, with some centres offering open access for the duration of the intervention, allowing participants to use the facilities outside of the two structured sessions per week.

*P14 “It was really… it has been marvellous and I’ve met so many people and it’s really lovely just to come in and feel the friendliness of it, that girl down at reception is wonderful.” F*

Specific to the SMS + ERS group, participants commented on the benefits associated with the components of the SMS intervention such as self-monitoring their behaviour and setting goals to reduce their sitting time and increase their activity levels. Participants stated that they recognised the importance of incorporating healthy behaviours into their routine and found it easier when alongside their peers in a supportive and reassuring environment.

*P24 “Certainly within the women, I felt that we were all supportive of each other. There was a lot of banter with the men but within the women I did feel that it was supportive and good fun.” F*

*P542 “The phone calls gave a little nudge. Like a reminder to do something.” M*

The subtheme of structural enablers included the perceived benefits of a structured programme and positive feedback on the variety of exercises included in the classes, specifically a preference for the circuit-based training component over the gym-based sessions due to the social element and aspect of enjoyment by completing the exercises as a group. It was evident from the discussions that enjoyment was a major influence on their overall experience, with music identified as a way of increasing enjoyment and enhancing mood. In addition, there was a general consensus regarding the importance training as a group.

*P42 “Coming to the group, very positive, because it gave me life.” M*

#### 3.1.3. Mechanisms of Impact

The overarching theme to emerge regarding mechanisms of impact was related to the participants’ views on how the SMS + ERS and ERS programme works. Four sub-themes emerged, including: (i) Habit formation, (ii) increased awareness of the health benefits associated with increasing PA and reducing SB, (iii) the impact of the lived experience of the programme on physical functioning and (iv) the impact of functional and emotional well-being in motivating change. Within the subtheme of habit formation, participants stated that their involvement in the SITLESS project had a positive influence on their behaviour, promoting self-motivation, which supported habit formation. Participants stated that their involvement in the programme motivated them to improve their health status and continue with their new healthier lifestyle behaviours.

*P37 “Well, I guess that you also make an effort and you see that it works/it does good to you, then it is silly to stop doing it. There are moments where you relax, and you see it was good to you, why not keep doing it? Then it is a motivation I have to keep doing what I learned. Because if it does good to me, why stop? Anyway, it is no effort…” M*

Specific to the SMS + ERS group, participants spoke of incorporating their new lifestyle into their routine. Participants described an increased appreciation of the benefits of setting goals and monitoring their activity and the motivation to sustain the healthier behaviours due to the positive impact it was having on their overall health.

*P11 “For me having the goals to work for really helped me and maybe that’s something to do with my personality but I really thought that concept was great.” F*

*P22 “It was when I started seeing results that I was able to get more specific with my goals. When people started commenting on my weight and I could notice my clothes getting looser and there was girls looking at me now who had never looked at me in the past [laughing] no seriously, when you see an improvement it keeps you motivated and it allowed me to set higher targets.” M*

Within the subtheme of increased awareness of the health benefits associated with increasing PA and reducing SB, participants described the influence that their participation in the programme had on other behaviours such as diet. They also described an increased awareness of the benefits associated with healthy behaviours and that their involvement in the programme positively influenced other lifestyle choices. Participants stated that their increased knowledge and understanding of the positive impact of reducing sitting time and increasing physical activity motivated them to sustain healthier lifestyle changes.

*P24 “I was actually surprised, when I became aware of how many hours I was sitting during the evening watching TV, I was quite shocked about how many hours I was sitting down.” M*

The subtheme surrounding the impact of the lived experience of the programme on physical functioning identified issues regarding a recognition of the participants’ own limitations, a motivation to improve their health and the positive relationship they developed with the trainer. Participants perceived improvements in mobility and, consequently, activities of daily living. They described improvements in their general and physical health and stated that their participation in the programme had a positive impact on their overall health.

*P37 “Physically better. It has helped me to… I don’t know…ahh to be physically better, although I’m one year older…”*

The final subtheme to emerge under mechanisms of impact was related to the impact of functional and emotional well-being which, in turn, motivated change. Participants acknowledged the perceived benefits of being part of a group and having that sense of belonging through social interaction led to the development of supportive networks, at least during the programme. Participants referred to the benefits associated with the social aspect of the programme and the importance of group dynamics in motivating participation. In addition, they described a sense of achievement with others, attributing a positive experience to the programme, including aspects of fun and meaningful learning.

*P593 “Significantly better well-being, I want to keep that as long as possible.” M*

#### 3.1.4. Perceived Effects

The overarching theme to emerge regarding perceived effects was related to the participants’ views on the outcomes resulting from the SMS + ERS and ERS programme. Three subthemes emerged from the SMS + ERS and ERS participant data, including: (i) Impact on social relationships, (ii) physical and mental well-being benefits of developing healthier behaviours and (iii) difficulties of maintaining change without supportive structures in place. Within the subtheme surrounding the impact on social relationships, participants referred to the development of new networks and social relationships and the benefits associated with sharing information and experiences with their peers. Participants referred to the physical and mental well-being benefits of developing healthier behaviours and specifically noted improvements in confidence and independence, physical well-being and emotional well-being as a result of participating in the SITLESS project.

*P31 “I can feel the effects on my hands, arms, legs and back. I can feel my muscles have grown and become stronger and I am maintaining these positive effects by training 3 times a week.” F*

*P32 “I called the trainer when I was at the hospital, as the doctor told me, that the only reason why I survived was because of my high level of physical health…” M*

A number of familiarities were identified from both intervention group participants in that they reflected positively on the exercise component and group-based training element. For those participants who were allocated to the SMS + ERS group, it was evident that they developed skills to support self-managing behaviours, perceiving an ease of integrating the SITLESS tips into their everyday life and activity routines. However, participants were in agreement across both groups regarding the difficulties in sustaining the positive lifestyle habits they had developed when the organised sessions ended. During the final follow-up interviews that were conducted, participants highlighted difficulties in maintaining change without supportive structures in place and acknowledged the barriers associated with self-managing behaviours without the trainer and/or group.

*P21 “It is difficult to maintain the healthy behaviour on your own, especially at our age, you need a lot of energy to get going, and I must admit that it’s getting more and more difficult.” M*

In particular, SMS + ERS group participants reported that the transferrable lessons from the SMS component were easier to incorporate long-term than lessons associated with the ERS programme alone. However, some participants of the ERS programme also incorporated some of the exercises as a routine to be performed at home.

*P34 “I am still very conscious of the goals and making sure I have targets that I need to reach and maintain.” F*

### 3.2. Control Group Findings (CTRL Participants)

Two themes were identified in relation to the control group participants’ experience of the SITLESS project. The two themes were (i) experience as a control group participant and (ii) response to being allocated to a control group. Regarding the ‘experience as a control group participant,’ two subthemes were identified surrounding the influence of receiving generic health advice and the importance of the health check and the benefits of receiving feedback. Participants stated that their involvement in the study resulted in an increased awareness and reinforcement of existing knowledge through the completion of the programme. Participants spoke of the benefits associated with carrying out the assessments and stated that it made them more conscious of their strengths and limitations. They recognised the importance of the regular assessment to monitor their functional ability and acknowledged that the assessment component alone could potentially act as a catalyst for change. The participants stated that completion of the questionnaires provoked thoughts on their current health status and that the assessment feedback provided personal information to prompt action.

*P37 “I liked to hear how I was doing and it was good to compare my results against the average for my age.” M*

*P25 “I thought they were great because they give me an idea of how fit or unfit that I was, so I didn’t mind them at all.” F*

Regarding the ‘response to being allocated to a control group,’ two subthemes were identified surrounding the impact on behaviour and the perceived effects associated with their involvement in the SITLESS project. Participants voiced disappointment in being allocated to the control group but reflected positively on the healthy living seminars and reported acceptability of the assessment components. In line with findings from the other groups, the participants spoke of implications associated with personal circumstances and current health status and the impact of time commitments and restraints (i.e., caring responsibilities) on their ability to be active. They also acknowledged the importance of supportive environments at home and the influence of family and friends on their behaviour, with reference made to seasonal influences such as dark evenings in the winter months and poor weather conditions in some of the countries making going for walks or being outdoors more difficult. Participants in the control group also acknowledged the importance of reducing sitting time and being more physically active, appreciating that sustained behaviour change requires additional support.

*P43 “I like that they [the SITLESS team] care for me.” F*

*P38 “I think the SITLESS programme in the background was motivating me actually to take more responsibility for my weight… no-one can do it for you and once you grasp that I think, that’s the trick. But again, knowing that you’re on a programme that’s very supportive is also a motivator and I’m very pleased looking at my results today that show my hard work has produced a good result. So I would say that even attending those two sessions even though I know what I should and shouldn’t be doing, did make me more aware.” M*

## 4. Discussion

The qualitative study presented is part of the process evaluation of the SITLESS clinical trial, which was conducted with mixed methods. The purpose of this study was to explore the experiences of older adults who took part in the SITLESS intervention to shed light on the complexities associated with older adults’ participation in theory-informed physical activity and sedentary behaviour programmes with the objective of identifying the strengths and limitations of the programmes from participants’ perspectives to inform future interventions and programme delivery.

The findings from this study demonstrated that the participants in the intervention groups (SMS + ERS and ERS) acknowledged the importance of peer support and social connectedness on their journey to increasing their activity. There was also a strong emphasis on the importance of the enjoyment aspect, evidenced in the social component, music incorporated into the sessions and the type of exercises included. The influence of enjoyment on the overall experience of exercise is well described in the literature. A recent review by Stevens et al. identified four sources of positive emotional responses to exercise [14], namely: (1) affective response—‘feeling-good’ during or immediately after exercise, (2) incidental affect—daily background mood and emotions that are not influenced by exercise, (3) affect processing—cognitively processing previous affective responses to exercise and (4) affectively charged motivational states—elicited through the pathways of intrinsic motivation, fear and hedonic motivation. SITLESS participants identified the social components of the programme and the environment they were conducted in (e.g., use of music) primarily influenced their enjoyment. This indicates that future exercise programmes for older adults should be designed to focus less on the enjoyment from exercise (affective response) and more on the experience, as this enjoyment of group social interactions may reframe barriers and provide motivation for participation to maintain their participation [15].

Participants perceived that physical activity increased their sense of purpose and self-belief and reported that they found the sessions important as they identified areas for improvement. The participants commented on a sense of achievement from the success of others and having a sense of belonging within their allocated groups. They stated that the regular group exercise sessions contributed to balanced health through social connectedness and mutual support. These findings are supported by a recent systematic review and meta-ethnography describing what influences physical activity in older adults and their experiences of physical activity [16]. The findings from this review mirror those from the current study, demonstrating that physical activity can help in regaining feelings of purpose, of being needed in collective group activity and by creating habitual routine and structure to the day. The findings suggest that in overcoming real and perceived barriers and by taking up or sustaining physical activities, older adults can further build self-esteem, all of which contributes to a fulfilling older age.

In terms of the perceived effects of the intervention, one of the most prolific findings was related to the positive impact of the programme on social relationships. Participants stated that the development of healthier behaviours led to improvements in aspects of physical and emotional well-being. Participants described improved exercise confidence and increased independence. They also stated that they perceived improvements in general mobility and had an increased capacity for activities of daily living. Overall, the majority of participants stated that the programme had a positive impact on their physical, emotional and social health. These findings are consistent with similar studies carried out in the older adult population, where key motivators regarding participation include perceived health benefits, peer support and social networking and key motivators concerning adherence include socialising, a sense of connectedness, expert guidance and a sense of dignity [17]. Furthermore, the findings from the current study support those from previous research conducted exploring older adults’ experiences of group-based physical activity from the GOAL trial [16]. The results from the GOAL trial provide insight into the benefits and challenges for older adults of exercising with their peers such as enhanced social connection, the enjoyment of challenging yet varied exercises and enhanced self-perceptions related to physical fitness, health and appearance. In addition, similar to the current study, health issues and familial responsibilities were identified as barriers to participation for some men and women. Participants perceived that group exercise also acted as a means of addressing social isolation and physical and mental health concerns [18].

Many of the participants reported enjoying taking part in a structured programme where specialists tailored the sessions at an appropriate level for them. For many of the participants, participation in exercise referral was a completely new experience, and they described feelings of apprehension and unfamiliarity with the layout of a fitness centre and how to use the complex equipment. They stated that these feelings subsided as they progressed through the programme. These findings are supported by Stathi and colleagues [19], who reported that participants described overcoming barriers such as embarrassment and fear of injury, with the exercise specialist appearing to have the most facilitating role in their transition into the new environment, highlighting the important role of the exercise specialist in ERS for older adults.

During the 12- and 18-month follow-up periods, participants acknowledged the difficulties in sustaining positive behaviours when the support mechanisms of the group and trainer were removed. Furthermore, other studies have also reported that participants voice disappointment when trials end [20,21,22]. Participants in the current study commented on the barriers of sustaining activity through a lack of social cohesiveness without the group and an awareness of a decline in their health through inactivity and sedentary behaviour.

There are a number of limitations in the study that should be noted. Given the intensive nature of qualitative research, we only included a subset of participants in these interviews and focus groups. Sample size per se is not a key consideration in qualitative research. Nevertheless, we sought to represent the full range of views from participants. In our study, the sample of participants was relatively large and from four different European countries, but it is of more relevance to highlight that the data gathered were rich in information to answer the research question and we achieved data saturation at each of the sites. Thus, we believe that we have accurately represented the views of participants. Finally, it was also not possible to offer an opportunity to participants to read a report of the findings for validation.

Focus groups were held, inviting all participants in the chosen natural groups (i.e., the exercise group they were involved in) at the same time and place where group-based intervention was conducted. This technique allowed researchers to easily reach a broad diversity of participants profiles in each arm, while interviews made it possible to select specific profiles of interest and explore more in-depth personal experiences in a confidential atmosphere. Triangulation of methods and informants was applied in this qualitative study with participant observation of the interventions and focus groups with trainers to strengthen the results. However, these components are not included in this manuscript. In this paper, only the qualitative findings from the research study are presented. Due to the wide scope of other data that have been collected, not all of our findings could be presented in one paper. Therefore, other analyses, as laid out in our published protocol [8], will be published in subsequent publications. Specifically, in the next steps of the SITLESS study, qualitative process evaluation results will be complemented with quantitative process evaluation results on the fidelity and adherence of the whole sample of participants (not yet published). Moreover, qualitative and quantitative process evaluation results will help to support the interpretation of impact evaluation outcomes (i.e., quantitative outcomes on effectiveness) (not yet published), thus reinforcing each other.

This paper has several implications for practice, research and policy. Regarding practice, the findings suggest that future programmes should be adapted in light of the evidence that many older adults participate in physical activity programmes for the social rewards and feelings of community engagement. Moreover, the current study also highlights the importance of offering continuation classes once programmes end to provide a continuity to motivated participants. Likewise, regarding policy, exit strategies such as commissioned maintenance programmes are important in ensuring the maintenance of physical activity and reduced sedentary behaviour. Last, in terms of research, future studies should focus on how physical activity can contribute to life satisfaction, sense of purpose and sense of role fulfilment in older age.

## 5. Conclusions

The findings from this study highlight the perceived barriers and facilitators to older adults’ engagement in exercise referral schemes. Evidence demonstrates that social interaction through the group-based programmes is a key component when designing interventions as they promote adherence and encourage the maintenance of targeted behaviours through peer support and connectedness. In addition, enjoyment represents a key aspect of a successful programme and a potential mediator of maintenance. In order to promote sustained levels of increased physical activity and reduced sedentary behaviour, exit strategies and signposting to relevant classes and facilities should be in place to facilitate the maintenance of positive lifestyle behaviours.

## Figures and Tables

**Table 1:** General information of the ERS and SMS interventions and the control group. Adapted from Giné-Garriga and colleagues [10].

Name of the Intervention Arm	Program Components	Training Responsible	Duration	General Structure of Each Session
ERS intervention	Aerobic training Strength-based/endurance exercises Balance-based functional exercises Flexibility exercises	Specially trained PA specialist: physical therapist; sport professional/trainer; ergotherapist with specific health qualification.	16 weeks Two sessions per week of 45–60 min The intervention was conducted in an indoor primary care, sports facility or municipality facilities (e.g., activity centres for older adults)	All training sessions began with a 5–10 min warm-up focussing on social and physical interactions, followed by 35 min of different exercises adapted to each individual’s functional level (according to the participants’ SPPB score *) All training sessions ended with cool-down (breathing exercises and stretching for 5–10 min
SMS intervention	Raising awareness on differences Associations, risks and benefits of SB and PA Setting personal activity goals (long-term achievement goals) Enhancing motivation Goal-setting focusing separately on SB and PA Self-monitoring (pedometer and activity diary) External monitoring (Instructor) Problem-solving according to the IDEA ** Social influence and social support Raising awareness on facilitators and barriers of PA and SB at home and neighbourhood Environmental signposting	The same specialist for the ERS intervention but trained and supervised on purpose to conduct the SMS intervention	A total of 7 sessions and 4 calls were conducted along 30 weeks: - 1 one-to-one session (week 1; 40 min) - 6 group-based sessions (weeks 3, 4, 5, 7, 9 and 11; 45–60 min) - 4 telephone calls (weeks 15, 20, 25 and 30; 20 min)	The SMS sessions included the following activities: (i) introducing the project to the participant, developing a rapport, (ii) setting a meaningful long-term goal to be achieved at the end of the intervention, (iii) identifying facilitators and barriers of PA and SB at home and neighbourhood in a group dynamic, (iv) environmental signposting to help engaging participants in local opportunities to do PA, (v) checking daily step counts registered in the activity diary and setting individual goals to increase steps or other physical activities, (vi) setting individual goals to reduce siting time set choosing recommendations (SITLESS tips) for decreasing SB, (vii) problem-solving techniques to overcome barriers to being less sedentary and more active according to the IDEA ** problem solving
Control arm	Health advice meetings	The same specialist for the ERS intervention	Two sessions of 45 min–1 h at week 5 and at week 11	Group-based talks with standardised topics about healthy lifestyle in the Primary Health Centre or the same setting where ERS takes place

* Total SPPB score ranges from 0 (worst performance) to 12 (best performance). Participants were classified into 3 different functional performance levels according to the results obtained: Low = 4–6; medium = 7–9; high functional level = 10–12 points. ** IDEA = Identifying the problem, developing a list of solutions, evaluating the solutions and analysing how the plan worked.

**Table 2:** Data collection summary.

Timepoint	SMS + ERS Participants	ERS Participants	CTRL Participants
Postintervention	8 × Focus Groups (n = 8 (6F), n = 5 (3F), n = 9 (4F), n = 4 (1F), n = 13 (10F), n = 11 (7F), n = 4 (1F), n = 4 (0F)	4 × Focus Groups (n = 7 (4F), n = 6 (5F), n = 6 (3F), n = 5 (1F)	6 × Interviews (3F)
	15 × Interviews (8F)		
12-month Follow-up	8 × Interviews (4F)	8 × Interviews (4F)	7 × Interviews (4F)
18-month Follow-up	8 × Interviews (5F)	8 × Interviews (4F)	7 × Interviews (4F)

Abbreviations: F = female.

**Table 3:** SITLESS matrix of findings: SMS + ERS and ERS group.

Framework	Overarching Theme	Subthemes	Categories															
Context	Environmental and personal factors that influence older adults experience of the SMS + ERS and ERS programme	Physical environmental factors	Availability of places to be active (proximity to their home)					Seasonal effect			Perceptions of fitness centres						Safety	
		Social environmental factors	Support at home					Caring responsibilities								Peer support		
		Personal factors	Health and well-being			Personality types and mood					Recognition of meaningful activity							
Implementation	Participants views on the components of the SMS+ERS and ERS programme	Social enablers	Personal enjoyment and satisfaction with the programme											Trainer				Peers
		Practical enablers	Self-monitoring (SMS specific)					Goal-setting (SMS specific)									Facilities	
		Structural enablers	Positive perception of group-based training				Exercise format						Music (mood enhancer)					
Mechanisms of Impact	Participants views on how the SMS+ERS and ERS programme works	Increased awareness of health benefits of ↑ PA and ↓ SB	Influence on other behaviours, i.e., dietary habits															
		Impact of lived experience of programme on physical functioning	Recognition of own limitations					Motivation to improve health					Positive relationship with trainer					
		Impact of functional and emotional well-being motivates change	Benefit associated with social aspect and group dynamics						Sense of achievement shared with others						Sense of belonging			
		Habit formation	Self-motivation	Incorporating new lifestyle into routine (SMS specific)														
Outcomes	Participants views on the outcomes resulting from the SMS+ERS and ERS programme	Impact on social relationships	Sharing information and experiences							Developing new networks								
		Physical and mental benefits of developing healthier behaviours	Confidence and independence		Physical well-being							Emotional well-being						
		Difficulties of maintaining change without supportive structures in place	Transferable lessons from the SMS component easier to incorporate long-term (SMS specific)															

**Table 4:** SITLESS matrix of findings: CTRL group.

Overarching Themes	Subthemes	
Experience as a CTRL group participant	Influence of receiving generic advice	Importance of health check and the benefits of receiving feedback
Response to being allocated to a CTRL group	Impact on behaviour	Perceived effects

## Data Availability

The data presented in this study are available on request from the corresponding author. The data are not publicly available at the time of publication due to ongoing analyses.

## Supplementary Materials

The following are available online at https://www.mdpi.com/article/10.3390/ijerph18094730/s1: Topic guides of the focus groups and interviews.
