# Supplementary material for: Older Adults’ Experiences of a Physical Activity and Sedentary Behaviour Intervention: A Nested Qualitative Study in the SITLESS Multi-Country Randomised Clinical Trial
Source: Int J Environ Res Public Health. 2021 Apr 29;18(9):4730. doi: 10.3390/ijerph18094730 (PMC8124427; doi:10.3390/ijerph18094730)
Supplement: Supplementary file 1 [file ijerph-18-04730-s001.zip › ijerph-1175121-supplementary.pdf]

SEMI-STRUCTURED QUESTIONNAIRE FOR THE FOCUS GROUP FOR THE SMS INTERVENTION GROUPS

Part one: introduction and presentation of participants (10')

Section	Comments
**Introduction**	• Acknowledgements<br>• The conductor introduces briefly him/her self to the participants<br>• Inform about the duration and the aims of the meeting<br>• The conductor informs the participants that the meeting will be audio-recorded for later analysis.<br>• The conductor reminds the participants to turn-off or silence phones.
**Informed consent**	• The conductor asks for permission about recording of the meeting and informed consent.<br>• Reading and signing of informed consent.
**Presentation of participants**	• Kindly ask participants to introduce their selves to the rest of the group.

Part two: overall mechanisms of impact (20')

Dimension	Questions
*According to participants' perceptions: how do participants' actively respond and interact with the **overall intervention**?*<br><br>**<u>Note:</u>** take into account that the SMS intervention requires following tasks:<br>○ *Fill the diary daily: steps or PA time, and calculate means.*<br>○ *Wear daily, open and read the pedometer.*<br>○ *Set goals on PA and follow-up accomplishment.*<br>○ *Set goals on SB and follow-up accomplishment (SITLESS tips).*<br>○ *Active participation in group discussions.*<br>○ *Telephone promots*	• What are your general impression / thoughts of the intervention?<br>• Overall how satisfied are you with the intervention?<br>• What specific features of the intervention impressed you?<br>• What features of the intervention disappointed you?<br>• How likely would you be to recommend this intervention to a family member or friend? Why?<br>• What, if anything, do you find frustrating or unappealing about the intervention?<br>• Do you have any suggestions on how we could improve the intervention to make it more appealing?<br>• Were any of tasks required in the intervention difficult for you to perform?

Part three: overall perceived effects and mechanisms of impact (20')

Dimension	Questions
**Secondary outcomes**	• Have you perceived any effects of the intervention? Which ones?<br>• Have you perceived any specific effects on<br>○ General health?<br>○<br>○ Mental health? In terms of depression, loneliness, etc. and psychological/emotional wellbeing?<br>○ Physical health?<br>○ Social relationships?
**Primary outcomes: Effects on PA and SB**<br><br>*How does the SMS intervention produce change?*	• During the intervention: have you increased your PA? (besides the ERS sessions)<br>• In case that you have increased your PA, why do you think you have done it?, Because your physical capacity increased<br>○ Because your self-efficacy increased<br>○ For other reasons<br>• In case that you have not increased your PA, why do you think you haven't done it?<br>• During the program: have you decreased your SB?<br>• If so, in which manner?<br>○ Incorporate new routines<br>○ Other<br>• If not, why do you think that?
**Mid-long term effects on PA and SB**	• Do you think you will maintain your increased PA / decreased SB once the intervention has finished?<br>• If so, in which manner?<br>• If not, why do you think that?

Part four: mechanisms of impact for each component (20’)

Dimension	Questions
*How do **each intervention components** contribute to produce change?* *Specifically, does the intervention work by improving self-efficacy and social support?* *Does a better accomplishment of the goal setting, self-monitoring and cues mediate the reduction of sedentary behaviour and the increase in physical activity?”*	- Can you state the strategies you have used to accomplish each session’s goals? Have you accomplished them by yourself? - What are the reasons why you have decided to accomplish (or not) the goals? - How and why each component supported/permitted to achieve the goals of the intervention? (mechanisms of impact) - Raising awareness on differences, associations, risks and benefits of SB and PA. - Setting personal activity goals (long-term achievement goals) - Goal setting focusing separately on PA - Goal setting focusing separately on SB (SITLESS tips) - Self-monitoring: - pedometer - activity diary - External monitoring (Instructor) - Problem-solving - Raising awareness on facilitators and barriers of PA and SB at home and at the neighborhood (environmental signposting) - Peer and social support from the group - The trainer - Telephone prompts

Part five: context (20’)

Dimension	Questions
**Physical environment**<br><br>*How do contextual factors regarding the characteristics of the neighbourhood affect outcomes according to participants’ perceptions?*	• How do you perceive the physical environment of your neighbourhood?<br>• How do you perceive the specific settings of the intervention? (i.e., facilities used for the SMS and PA sessions)<br>○ Primary health care centre<br>○ Parks<br>○ Sports facilities<br>○ Community resources<br>• How do you perceive these specific aspects of your neighbourhood? (especially considering whether and how these aspects affect doing PA in the neighbourhood)<br>○ Socio-economic level<br>○ Urban/rural setting<br>○ Orography / walkability<br>○ Pleasant environment (comfortable, traffic…)<br>○ Personal safety (crimes, robberies, …)<br>○ Availability of places within an easy walking distance to do informal PA (parks, urban gym, …) and to do formal PA (organized PA offers)
**Personal networks**<br><br>*How do contextual factors regarding the characteristics of the personal network affect outcomes according to participants’ perceptions?*	• Do you think your personal situation or health status (e.g., having a supportive social network, being a caregiver, living alone, having a chronic disease, etc.) helps or hinders your physical activity and your sedentary behavior? How and why?<br>• Do you think your personal network (partners, family, friends) helps or hinders your physical activity and your sedentary behavior? How and why?

	• Did you know other participants of the group before? How has that affected your participation in the group?

SEMI-STRUCTURED QUESTIONNAIRE FOR INTERVIEWS WITH PARTICIPANTS OF THE SMS GROUP

Note: Aspects related with gender, frailty/robustness and ethnicity have been considered as elegibility criteria and will be considered in the content analysis. However, this questionnaire does not include any specific question regarding these aspects and are considered to emerge in their discourse.

Part one: introduction (5')

Section	Comments
**Introduction**	• Acknowledgements<br>• The conductor introduces briefly him/her self to the participant<br>• Inform about the duration and the aims of the meeting<br>• The conductor informs the participant that the meeting will be audio-recorded for later analysis.<br>• The conductor reminds the participant to turn-off or silence the phone.
**Informed consent**	• The conductor asks for permission about recording of the interview and informed consent.<br>• Reading and signing of informed consent.

Part two: overall mechanisms of impact (20')

Dimension	Questions
*According to participant' perceptions: how do the participant' actively respond and interact with the **overall intervention**?*<br><br>__Note:__ take into account that the SMS intervention requires following tasks:<br>◦ *Fill the diary daily: steps or PA time, and calculate means.*<br>◦ *Wear daily, open and read the pedometer.*<br>◦ *Set goals on PA and follow-up accomplishment.*<br>◦ *Set goals on SB and follow-up accomplishment (SITLESS tips).*<br>◦ *Active participation in group discussions.*<br>◦ *Telephone prompts*	• What are your general impression / thoughts of the intervention?<br>• Overall how satisfied are you with the intervention?<br>• What specific features of the intervention impressed you?<br>• What features of the intervention disappointed you?<br>• How likely would you be to recommend this intervention to a family member or friend? Why?<br>• What, if anything, do you find frustrating or unappealing about the intervention?<br>• Do you have any suggestions on how we could improve the intervention to make it more appealing?<br>• Were any of tasks required in the intervention difficult for you to perform?

Part three: overall perceived effects and mechanisms of impact (20')

Dimension	Questions
**Secondary outcomes**	• Have you perceived any effects of the intervention? Which ones?<br>• Have you perceived any specific effects on<br>○ General health?<br>○ Mental health? In terms of depression, loneliness, etc. and psychological/emotional wellbeing?<br>○ Physical health?<br>○ Social relationships?
**Primary outcomes: Effects on PA and SB**<br><br>*How does the SMS intervention produce change?*	• During the intervention: have you increased your PA? (besides the ERS sessions)<br>• In case that you have increased your PA, why do you think you have done it?, Because your physical capacity increased<br>○ Because your self-efficacy increased<br>○ For other reasons<br>• In case that you have not increased your PA, why do you think you haven't done it?<br>• During the program: have you decreased your SB?<br>• If so, in which manner?<br>○ Incorporate new routines<br>○ Other<br>• If not, why do you think that?
**Mid-long term effects on PA and SB**	• Do you think you will maintain your increased PA / decreased SB once the intervention has finished?<br>• If so, in which manner?<br>• If not, why do you think that?

Part four: mechanisms of impact for each component (20')

Dimension	Questions
▸ *How do **each intervention components** contribute to produce change?*<br>▸ *Specifically, does the intervention work by improving self-efficacy and social support?*<br>▸ *Does a better accomplishment of the goal setting, self-monitoring and cues mediate the reduction of sedentary behaviour and the increase in physical activity?"*	• Can you state the strategies you have used to accomplish each session's goals? Have you accomplished them by yourself?<br>• What are the reasons why you have decided to accomplish (or not) the goals?<br>• How and why each component supported/permitted to achieve the goals of the intervention? (mechanisms of impact)<br>○ Raising awareness on differences, associations, risks and benefits of SB and PA.<br>○ Setting personal activity goals (long-term achievement goals)<br>○ Goal setting focusing separately on PA<br>○ Goal setting focusing separately on SB (SITLESS tips)<br>○ Self-monitoring:<br>▪ pedometer<br>▪ activity diary<br>○ External monitoring (Instructor)<br>○ Problem-solving<br>○ Raising awareness on facilitators and barriers of PA and SB at home and at the neighborhood (environmental signposting)<br>○ Peer and social support from the group<br>○ The trainer<br>○ Telephone prompts

Part five: context (20’)

Dimension	Questions
**Physical environment**<br><br>*How do contextual factors regarding the characteristics of the neighbourhood affect outcomes according to participants’ perceptions?*	• How do you perceive the physical environment of your neighbourhood?<br>• How do you perceive the specific settings of the intervention? (i.e., facilities used for the SMS and PA sessions)<br>○ Primary health care centre<br>○ Parks<br>○ Sports facilities<br>○ Community resources<br>• How do you perceive these specific aspects of your neighbourhood? (especially considering whether and how these aspects affect doing PA in the neighbourhood)<br>○ Socio-economic level<br>○ Urban/rural setting<br>○ Orography / walkability<br>○ Pleasant environment (comfortable, traffic…)<br>○ Personal safety (crimes, robberies, …)<br>○ Availability of places within an easy walking distance to do informal PA (parks, urban gym, …) and to do formal PA (organized PA offers)
**Personal networks**<br><br>*How do contextual factors regarding the characteristics of the personal network affect outcomes according to the participant’s perceptions?*	• Do you think your personal situation or health status (e.g., having a supportive social network, being a caregiver, living alone, having a chronic disease, etc.) helps or hinders your physical activity and your sedentary behavior? How and why?<br>• Do you think your personal network (partners, family, friends) helps or hinders your physical activity and your sedentary behavior? How and why?

	• Did you know other participants of the group before? How has that affected your participation in the group?

SEMI-STRUCTURED QUESTIONNAIRE FOR THE FOCUS GROUP FOR THE ONLY PA INTERVENTION GROUPS

Part one: introduction and presentation of participants (10')

Section	Comments
**Introduction**	• Acknowledgements<br>• The conductor introduces briefly him/her self to the participants<br>• Inform about the duration and the aims of the meeting<br>• The conductor informs the participants that the meeting will be audio-recorded for later analysis.<br>• The conductor reminds the participants to turn-off or silence phones.
**Informed consent**	• The conductor asks for permission about recording of the meeting and informed consent.<br>• Reading and signing of informed consent.
**Presentation of participants**	• Kindly ask participants to introduce their selves to the rest of the group.

Part two: overall mechanisms of impact (20’)

Dimension	Questions
*According to participants’ perceptions: how do participants’ actively respond and interact with the **PA intervention**?*	• What are your general impression / thoughts of the intervention?<br>• Overall, how satisfied are you with the intervention?<br>• What specific features of the intervention impressed you?<br>• What features of the intervention disappointed you?<br>• How likely would you be to recommend this intervention to a family member or friend? Why?<br>• What, if anything, do you find frustrating or unappealing about the intervention?<br>• Do you have any suggestions on how we could improve the intervention to make it more appealing?<br>• Were any of tasks required in the intervention difficult for you to perform?

Part three: overall perceived effects and mechanisms of impact (20')

Dimension	Questions
**Secondary outcomes**	• Have you perceived any effects of the intervention? Which ones?<br>• Have you perceived any specific effects on<br>o General health?<br>o Mental health? In terms of depression, loneliness, etc. and psychological/emotional wellbeing?<br>o Physical health?<br>o Social relationships?
**Primary outcomes: Effects on PA and SB**<br><br>*How does the PA intervention produce change? (mechanisms of impact)*	• During the intervention: have you increased your PA? (besides the ERS sessions)<br>• In case that you have increased your PA, why do you think you have done it?,<br>- Because your self-efficacy increased<br>- Because your physical capacity increased<br>- Peer support from other members of the group (e.g., walks together outside the group…)<br>- Sense of belonging to the group<br>- The trainer (professional support)<br>- For other reasons<br>• In case that you have not increased your PA, why do you think you haven't done it?<br>• During the program: have you decreased your SB?<br>• If so, in which manner?<br>o Incorporate new routines<br>o Other<br>• If not, why do you think that?
**Mid-long term effects on PA and SB**	• Do you think you will maintain your increased PA / decreased SB once the intervention has finished?<br>• If so, in which manner?<br>• If not, why do you think that?

Part five: context (20')

Dimension	Questions
**Physical environment**<br><br>*How do contextual factors regarding the characteristics of the neighbourhood affect outcomes according to participants' perceptions?*	• How do you perceive the physical environment of your neighbourhood?<br>• How do you perceive the specific settings of the intervention? (i.e., facilities used for the SMS and PA sessions)<br>○ Primary health care centre<br>○ Parks<br>○ Sports facilities<br>○ Community resources<br>• How do you perceive these specific aspects of your neighbourhood? (especially considering whether and how these aspects affect doing PA in the neighbourhood)<br>○ Socio-economic level<br>○ Urban/rural setting<br>○ Orography / walkability<br>○ Pleasant environment (comfortable, traffic…)<br>○ Personal safety (crimes, robberies, …)<br>○ Availability of places within an easy walking distance to do informal PA (parks, urban gym, …) and to do formal PA (organized PA offers)<br>• How do you think the physical environment of your neighbourhood helps or hinders to achieve the intervention aims?
**Personal networks**<br><br>*How do contextual factors regarding the characteristics of the personal network affect outcomes according to participants' perceptions?*	• Do you think your personal situation or health status (e.g., having a supportive social network, being a caregiver, living alone, having a chronic disease, etc.) helps or hinders your physical activity and your sedentary behavior? How and why?<br>• Do you think your personal network (partners, family, friends) helps or hinders your physical activity and your sedentary behavior? How and why?

	• Did you know other participants of the group before? How has that affected your participation in the group?

SEMI-STRUCTURED QUESTIONNAIRE FOR THE INTERVIEWS WITH THE PARTICIPANTS OF THE CONTROL GROUP

Part one: introduction and presentation of participants (10')

Section	Comments
**Introduction**	• Acknowledgements<br>• The conductor introduces briefly him/her self to the participant<br>• Inform about the duration and the aims of the meeting<br>• The conductor informs the participant that the meeting will be audio-recorded for later analysis.<br>• The conductor reminds the participant to turn-off or silence phones.
**Informed consent**	• The conductor asks for permission about recording of the meeting and informed consent.<br>• Reading and signing of informed consent.
**Presentation of participants**	• Kindly ask the participant to introduce themself.

Part two: overall mechanisms of impact (20')

Dimension	Questions
According to participants' perceptions: how do participants of the control group experience their participation in the Sitless project?	• How did you get involved in the SITESS project?<br>• What motivated you to agree to participate? What did you expect?<br>• How was your experience during this period of time?<br>• Have you attended the two sessions about healthy habits? If so, which was your experience with the healthy habits sessions?<br>• Which was your experience with the assessments (including the actigraph)?

Part three: overall perceived effects and mechanisms of impact (20’)

Dimension	Questions
**Secondary outcomes**	• During this period of time: have you experienced any changes in your health? If so, which ones? Due to the healthy habits sessions? Due to the engagement in the SITLESS project?<br>• Specifically, have you experienced any specific changes on:<br>○ General health?<br>○ Mental health? In terms of depression, loneliness, etc. and psychological/emotional wellbeing?<br>○ Physical health?<br>○ Social relationships?
**Primary outcomes: Effects on PA and SB**<br><br>*Whether and, if so, how does the healthy habits sessions produce change? (mechanisms of impact)*	• During this period of time: have you increased your **PA**? How and why? Due to the healthy habits sessions? Due to the engagement in the SITLESS project?<br>• In case that you have not increased your PA, why do you think you haven’t done it?<br>• During this period of time: have you decreased your **SB**?<br>• If so, in which manner?<br>○ Incorporate new routines<br>○ Other<br>• If not, why do you think that?<br>• Do you think you will maintain your increased PA / decreased SB?<br>• If so, in which manner?<br>• If not, why do you think that?

Part five: context (20’)

Dimension	Questions
**Physical environment**<br><br>*How do contextual factors regarding the characteristics of the neighbourhood affect outcomes*	• How do you perceive the physical environment of your neighbourhood?<br>• How do you perceive these specific aspects of your neighbourhood? (especially considering whether and how these aspects affect doing PA in the neighbourhood)<br>○ Socio-economic level

*(*physical activity or sedentary behaviour) *according to participants' perceptions?*	○ Urban/rural setting<br>○ Orography / walkability<br>○ Pleasant environment (comfortable, traffic…)<br>○ Personal safety (crimes, robberies, …)<br>○ Availability of places within an easy walking distance to do informal PA (parks, urban gym, …) and to do formal PA (organized PA offers)<br>• How do you think the physical environment of your neighbourhood helps or hinders your physical activity and your sedentary behavior?
**Personal networks**<br><br>*How do the personal situation and the personal network affect outcomes (*physical activity or sedentary behaviour) *according to participants' perceptions?*	• Do you think your personal situation or health status (e.g., having a supportive social network, being a caregiver, living alone, having a chronic disease, etc.) helps or hinders your physical activity and your sedentary behavior? How and why?<br>• Do you think your personal network (partners, family, friends) helps or hinders your physical activity and your sedentary behavior? How and why?

SEMI-STRUCTURED QUESTIONNAIRE FOR INTERVIEWS WITH PARTICIPANTS OF THE SMS GROUP 12 and the 18 MONTHS FOLLOW-UP

Note: Aspects related with gender, frailty/robustness and ethnicity have been considered as eligibility criteria and will be considered in the content analysis. However, this questionnaire does not include any specific question regarding these aspects and are considered to emerge in their discourse.

Part one: introduction (5')

Section	Comments
**Introduction**	• Acknowledgements<br>• The conductor introduces briefly him/herself to the participant<br>• Inform about the duration and the aims of the meeting<br>• The conductor informs the participant that the meeting will be audio-recorded for later analysis.<br>• The conductor reminds the participant to turn-off or silence the phone.
**Informed consent**	• The conductor asks for permission about recording of the interview and informed consent.<br>• Reading and signing of informed consent.

Part two: overall mechanisms of impact (20')

<table>
<tr><th>Dimension</th><th>Questions</th></tr>
<tr><td>

According to participant' perceptions: how do the participant' actively respond and interact with the ***overall intervention****?*

<u>Note:</u> Take into account that after the face-to face SMS intervention, the participant kept and could further use the diary, kept the pedometer and received the fours telephone prompts:

- *Diary.*
- *Pedometer.*
- *Telephone prompts*

</td><td>

- One year ago, you finished the SITLESS intervention, what was your general impression / thoughts of the intervention?
- Overall how satisfied were you with the intervention?
- Are there specific features of the intervention that you continue doing (diary, pedometer)?
- What, if anything, did you find frustrating or unappealing about the intervention?
- Were the telephone prompts helpful?
- Were any of tasks suggested in the intervention difficult for you to perform? And difficult to maintain during this year after the intervention?
- Do you have any suggestions on how we could improve the post-intervention period to make easier to maintain an increase on PA and/or a reduction on SB?

</td></tr>
</table>

Part three: overall perceived effects and mechanisms of impact (20')

Dimension	Questions
**Secondary outcomes (short and mid-long term effects)**	• Did you perceive any effects of the intervention? Which ones? Which effects have lasted?<br>• Have you perceived any specific effects on:<br>○ General health?<br>○ Mental health? In terms of depression, loneliness, etc. and psychological/emotional wellbeing?<br>○ Physical health?<br>○ Social relationships?
**Primary outcomes: Effects on PA and SB (short and mid-long term effects)**<br><br>*How does the SMS+ERS intervention produce change?*	• During the intervention: did you increase your PA? (besides the ERS sessions) Did this increase lasted during this year?<br>• In case that you have increased your PA, why do you think you have done it? Why did it last?<br>○ Because your physical capacity increased<br>○ Because your self-efficacy increased<br>○ Motivation to increase your PA before the intervention<br>○ For other reasons<br>• In case that you have not increased your PA, why do you think you haven't done it?<br>• During the program: did you decrease your SB? Did this decrease last during this year?<br>• If so, in which manner?<br>○ Incorporate new routines<br>○ Other (volume, intensity, frequency…)<br>• If not, why do you think it was this way?

Part four: mechanisms of impact for each component (20')

Dimension	Questions
*How do **each intervention components** contribute to produce change?* *Specifically, does the intervention work by improving self-efficacy and social support?* *Does a better accomplishment of the goal setting, self-monitoring and cues mediate the reduction of sedentary behaviour and the increase in physical activity?"*	- Can you state the strategies you have used to accomplish each session's goals? Have you accomplished them by yourself? - What are the reasons why you have decided to accomplish (or not) the goals? - How and why each component supported/permitted to achieve the goals of the intervention? (mechanisms of impact) - Raising awareness on differences, associations, risks and benefits of SB and PA. - Setting personal activity goals (long-term achievement goals) - Goal setting focusing separately on PA - Goal setting focusing separately on SB (SITLESS tips) - Self-monitoring: - pedometer - activity diary - External monitoring (Instructor) - Problem-solving - Raising awareness on facilitators and barriers of PA and SB at home and at the neighborhood (environmental signposting) - Peer and social support from the group - The trainer - Telephone prompts

Part five: context (20’)

Dimension	Questions
**Physical environment**<br><br>*How do contextual factors regarding the characteristics of the neighbourhood affect outcomes according to participants’ perceptions?*	• How do you perceive the physical environment of your neighbourhood?<br>• How do you perceive the specific settings of the intervention? (i.e., facilities used for the SMS and PA sessions)<br>○ Primary health care centre<br>○ Parks<br>○ Sports facilities<br>○ Community resources<br>• Have you started activities in your neighbourhood since the end of the intervention?<br>• How do you perceive following specific aspects of your neighbourhood? (especially considering whether and how these aspects affect doing PA in the neighbourhood)<br>○ Socio-economic level<br>○ Urban/rural setting<br>○ Orography / walkability<br>○ Pleasant environment (comfortable, traffic…)<br>○ Personal safety (crimes, robberies, …)<br>○ Availability of places within an easy walking distance to do informal PA (parks, urban gym, …) and to do formal PA (organized PA offers)
**Personal networks**<br><br>*How do contextual factors regarding the characteristics of the personal network affect outcomes according to the participant’s perceptions?*	• Do you think your personal situation or health status (e.g., having a supportive social network, being a caregiver, living alone, having a chronic disease, etc.) helps or hinders your physical activity and your sedentary behavior? How and why? (During the intervention and during this last year)<br>• Do you think your personal network (partners, family, friends) helps or hinders your physical

	activity and your sedentary behavior? How and why? (During the intervention and during this last year)<br>• Did you know other participants of the group before? Did you keep in touch with the other participants of the group? Did you conduct PA together or other social activities?

SEMI-STRUCTURED QUESTIONNAIRE FOR INTERVIEWS WITH PARTICIPANTS OF THE ERS GROUP 12 and the 18 MONTHS FOLLOW-UP

Part one: introduction and presentation of participants (10')

Section	Comments
**Introduction**	• Acknowledgements<br>• The conductor introduces briefly him/herself to the participant<br>• Inform about the duration and the aims of the meeting<br>• The conductor informs the participant that the meeting will be audio-recorded for later analysis.<br>• The conductor reminds the participant to turn-off or silence the phone.
**Informed consent**	• The conductor asks for permission about recording of the interview and informed consent.<br>• Reading and signing of informed consent.

Part two: overall mechanisms of impact (20')

Dimension	Questions
*According to participants' perceptions: how do participants' actively respond and interact with the **PA intervention**?*	• One year ago, you finished the SITLESS intervention, what was your general impression / thoughts of the intervention?<br>• Overall how satisfied were you with the intervention?<br>• Are there specific exercises of the intervention that you continue doing?<br>• What, if anything, did you find frustrating or unappealing about the intervention?<br>• Were any of tasks suggested in the intervention difficult for you to perform? And difficult to maintain during this year after the intervention?<br>• Do you have any suggestions on how we could improve the post-intervention period to make easier to maintain an increase on PA and/or a reduction on SB?

Part three: overall perceived effects and mechanisms of impact (20')

Dimension	Questions
**Secondary outcomes (short and mid-long term effects)**	• Did you perceive any effects of the intervention? Which ones? Which effects have lasted?<br>• Have you perceived any specific effects on:<br>○ General health?<br>○ Mental health? In terms of depression, loneliness, etc. and psychological/emotional wellbeing?<br>○ Physical health?<br>○ Social relationships?
**Primary outcomes: Effects on PA and SB (short and mid-long term effects)**<br><br>*How does the ERS intervention produce change?*	• During the intervention: did you increase your PA? (besides the ERS sessions) Did this increase lasted during this year?<br>• In case that you have increased your PA, why do you think you have done it? Why did it last?<br>○ Because your physical capacity increased<br>○ Because your self-efficacy increased<br>○ Motivation to increase your PA before the intervention<br>○ For other reasons<br>• In case that you have not increased your PA, why do you think you haven't done it?<br>• During the program: did you decrease your SB? Did this decrease last during this year?<br>• If so, in which manner?<br>○ Incorporate new routines<br>○ Other (volume, intensity, frequency…)<br>• If not, why do you think that?

Part five: context (20')

Dimension	Questions
**Physical environment**<br><br>*How do contextual factors regarding the characteristics of the neighbourhood affect outcomes according to participants' perceptions?*	• How do you perceive the physical environment of your neighbourhood?<br>• How do you perceive the specific settings of the intervention? (i.e., facilities used for the SMS and PA sessions)<br>○ Primary health care centre<br>○ Parks<br>○ Sports facilities<br>○ Community resources<br>• Have you started activities in your neighbourhood since the end of the intervention?<br>• How do you perceive following specific aspects of your neighbourhood? (especially considering whether and how these aspects affect doing PA in the neighbourhood)<br>○ Socio-economic level<br>○ Urban/rural setting<br>○ Orography / walkability<br>○ Pleasant environment (comfortable, traffic…)<br>○ Personal safety (crimes, robberies, …)<br>○ Availability of places within an easy walking distance to do informal PA (parks, urban gym, …) and to do formal PA (organized PA offers)
**Personal networks**<br><br>*How do contextual factors regarding the characteristics of the personal network affect outcomes according to participants' perceptions?*	• Do you think your personal situation or health status (e.g., having a supportive social network, being a caregiver, living alone, having a chronic disease, etc.) helps or hinders your physical activity and your sedentary behavior? How and why? (During the intervention and during this last year)

	• Do you think your personal network (partners, family, friends) helps or hinders your physical activity and your sedentary behavior? How and why? (During the intervention and during this last year)<br>• Did you know other participants of the group before? Did you keep in touch with the other participants of the group? Did you conduct PA together or other social activities?

SEMI-STRUCTURED QUESTIONNAIRE FOR INTERVIEWS WITH PARTICIPANTS OF THE CONTROL GROUP 12 and the 18 MONTHS FOLLOW-UP

Part one: introduction and presentation of participants (10')

Section	Comments
**Introduction**	• Acknowledgements<br>• The conductor introduces briefly him/herself to the participant<br>• Inform about the duration and the aims of the meeting<br>• The conductor informs the participant that the meeting will be audio-recorded for later analysis.<br>• The conductor reminds the participant to turn-off or silence the phone.
**Informed consent**	• The conductor asks for permission about recording of the interview and informed consent.<br>• Reading and signing of informed consent.

Part two: overall mechanisms of impact (20')

Dimension	Questions
According to participants' perceptions: how do participants of the control group experience their participation in the Sitless project?	• How did you get involved in the SITESS project?<br>• What motivated you to agree to participate? What did you expect?<br>• How was your experience during this period of time?<br>• Did you attended the two sessions about healthy habits? If so, which was your experience with the healthy habits sessions?<br>• Which was your experience with the assessments (including the actigraph)?

Part three: overall perceived effects and mechanisms of impact (20')

Dimension	Questions
**Secondary outcomes**	• During this period of time: have you experienced any changes in your health? If so, which ones? Due to the healthy habits sessions? Due to the engagement in the SITLESS project?<br>• Specifically, have you experienced any specific changes on:<br>○ General health?<br>○ Mental health? In terms of depression, loneliness, etc. and psychological/emotional wellbeing?<br>○ Physical health?<br>○ Social relationships?
**Primary outcomes: Effects on PA and SB**<br><br>*Whether and, if so, how does the healthy habits sessions produce change? (mechanisms of impact)*	• During this period of time: have you increased your **PA**? How and why? Due to the healthy habits sessions? Due to the engagement in the SITLESS project?<br>• In case that you have not increased your PA, why do you think you haven't done it?<br>• During this period of time: have you decreased your **SB**?<br>• If so, in which manner?<br>○ Incorporate new routines<br>○ Other<br>• If not, why do you think that?<br>• Do you think you will maintain your increased PA / decreased SB?<br>• If so, in which manner?<br>• If not, why do you think that?

Part five: context (20')

Dimension	Questions
**Physical environment**<br><br>*How do contextual factors regarding the characteristics of the neighbourhood affect outcomes (*physical activity or sedentary behaviour) *according to participants' perceptions?*	• How do you perceive the physical environment of your neighbourhood?<br>• How do you perceive these specific aspects of your neighbourhood? (especially considering whether and how these aspects affect doing PA in the neighbourhood)<br>○ Socio-economic level<br>○ Urban/rural setting<br>○ Orography / walkability<br>○ Pleasant environment (comfortable, traffic…)<br>○ Personal safety (crimes, robberies, …)<br>○ Availability of places within an easy walking distance to do informal PA (parks, urban gym, …) and to do formal PA (organized PA offers)<br>• How do you think the physical environment of your neighbourhood helps or hinders your physical activity and your sedentary behavior?
**Personal networks**<br><br>*How do the personal situation and the personal network affect outcomes (*physical activity or sedentary behaviour) *according to participants' perceptions?*	• Do you think your personal situation or health status (e.g., having a supportive social network, being a caregiver, living alone, having a chronic disease, etc.) helps or hinders your physical activity and your sedentary behavior? How and why?<br>• Do you think your personal network (partners, family, friends) helps or hinders your physical activity and your sedentary behavior? How and why?
